# Mutation of a conserved lysine in the kinase homology domain reduces the natriuretic peptide-dependent activity and phosphorylation of guanylyl cyclase-A

**DOI:** 10.1186/2050-6511-16-S1-A47

**Published:** 2015-09-02

**Authors:** Aaron B Edmund, Lincoln R Potter

**Affiliations:** 1 Department of Biochemistry, Molecular Biology and Biophysics, University of Minnesota, Minneapolis, MN, USA

## Background and Results

**Figure 1:**
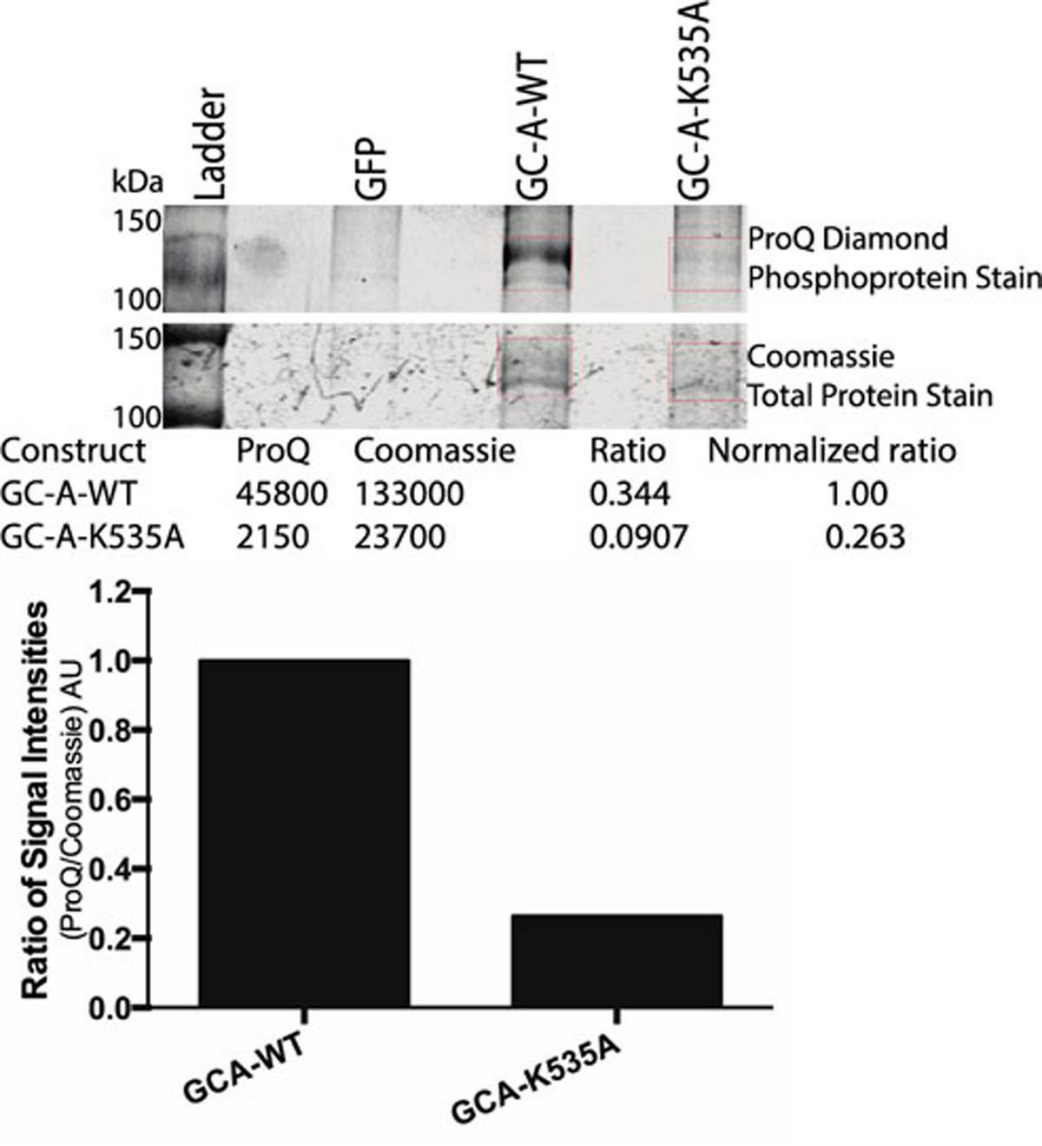
ProQ Diamond staining of phosphate concentrations and Coomassie staining of protein concentrations of wild type GC-A and K535A-GC-A purified from transiently transfected 293T cells by sequential immunoprecipitation and SDS-PAGE fractionation. Green fluorescent protein (GFP) is a negative control for the immunoprecipitation of GC-A.

Atrial natriuretic peptide (ANP) and B-type natriuretic peptide (BNP) regulate the cardiovascular system and metabolism by activating the membrane-spanning enzyme, guanylyl cyclase-A (GC-A) [1]. Eight serine and threonine phosphorylation sites have been identified between the membrane-spanning region and the beginning of the kinase homology domain (KHD) of GC-A [2,3]. Mutation of four or more of these sites to alanine produced a mutant form of GC-A that could not be activated by ANP, and prolonged exposure to ANP or acute exposure to protein kinase C activators resulted in the dephosphorylation and inactivation of GC-A [4,5]. Despite the clear role of phosphorylation in the regulation of GC-A, it is not known how it is phosphorylated or whether the KHD has intrinsic protein kinase activity. A previous study demonstrated that the K535A missense mutant of GC-A lacks ANP-dependent guanylyl cyclase activity while maintaining a functional catalytic domain, which is consistent with the mutation reducing GC-A phosphorylation [6]. K535 corresponds to an invariant lysine in known protein kinases that forms a salt bridge that stabilizes the N-terminal small lobe of the kinase. In some structures this lysine also interacts with the alpha and beta phosphates of ATP to facilitate substrate positioning. Here, we show first measurements of GC-A protein concentrations based on Coomassie staining and GC-A phosphate concentrations based on ProQ Diamond staining of SDS gels containing immunopurified GC-A to estimate the stoichiometry of the phosphorylation of the K535A mutant compared to wild type GC-A (Figure 1). The initial experiment indicates that K535A-GC-A has a reduced phosphorylation to protein ratio. Additional experiments will be optimized to increase signal to noise and repeated multiple times to determine if the reduced phosphorylation state of the K535A mutant is significantly less than that measured for wild type GC-A.
